# Histopathological, immunohistochemical and molecular biologic study of an enucleated specimen of a case of Eales’ disease

**DOI:** 10.1186/s12348-021-00259-x

**Published:** 2021-09-01

**Authors:** Amravi Shah, Sneha Giridhar, Gazal Patnaik, Radhika Mhatre, Dipankar Das, M. K. Janani, Anita Mahadevan, Jyotirmay Biswas

**Affiliations:** 1 Uvea Department, Medical Research Foundation, Sankara Nethralaya, 18 College Road, Tamilnadu 600006 Chennai, India; 2 Shri BhagwanMahavir Vitreoretinal Services Sankara Nethralaya, 18 College Road, Tamilnadu Chennai, India; 3 Department of Neuropathology, NIMHANS, Bengaluru, Karnataka India; 4 Department of Uvea, Sri SankaradevaNethralaya, Guwahati, Assam India; 5 Sankara Nethralaya Referral Laboratory, Tamil Nadu Chennai, India

**Keywords:** Eales’, Immunohistochemistry, PCR, Retinal, Vasculitis

## Abstract

Eales’ disease is a retinal vasculitis characterized by retinal inflammation, ischemia, and neovascularization. Exact pathogenesis of this disease is yet to be found out. We present a 29-year-old male, diagnosed with Eales’ disease in both eyes with persistent intraocular inflammation. Enucleation of the pthisical right eye was subjected for histopathological examination immunohistochemistry and molecular biologic study for mycobacterial tuberculosis DNA. Our study showed that Eales disease is probably a T cell mediated disease which is triggered by mycobacterial TB DNA. Further studies are needed to confirm our findings.

## Introduction

Eales’ disease is a retinal vasculitis predominantly affecting the peripheral retina of young and otherwise healthy adults [1]. Etiopathogenesis of this disease is still not clear [1]. Recent molecular biologics studies have shown mycobacterium tuberculosis DNA in Eales disease specimens [2]. Histopathological study of Eales disease eye has rarely been done. We report histopathologic, immunohistochemistry (IHC) and molecular biologic study of an enucleated specimen of a case of Eales’ disease.

## Case report

**Fig. 1:**
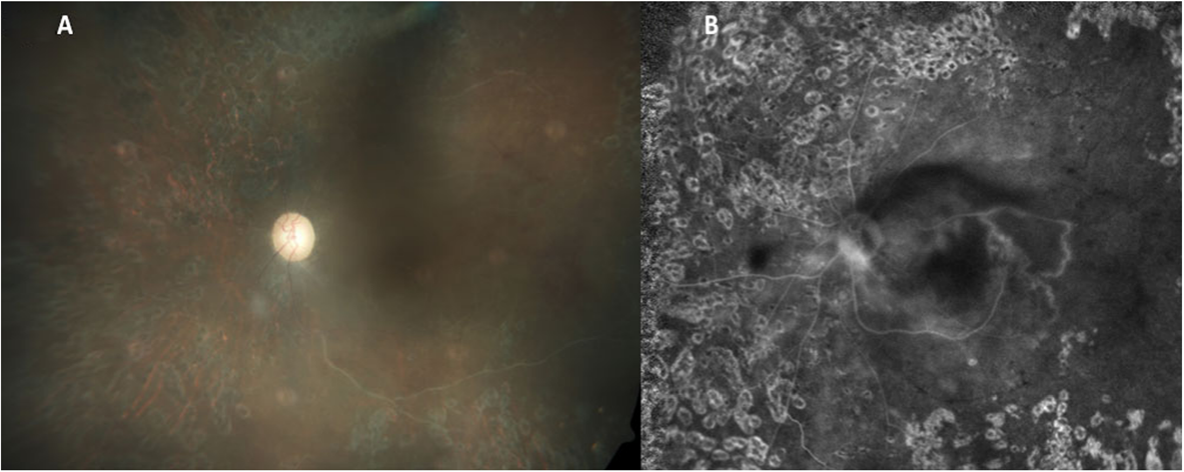
**A** Clinical photograph of left eye fundus showing media haze at the posterior pole, a pale optic disc with attenuated and sclerosed vessels. There are few retinal hemorrhages along with collaterals temporal to the macula. Mid-peripheral retina shows laser photocoagulation scars. **B**: Fundus fluorescein angiography in the arterio-venous phase showing disc leakage with active vasculitis temporal to fovea. Ischemic areas are also noted temporal to macula

**Fig. 2:**
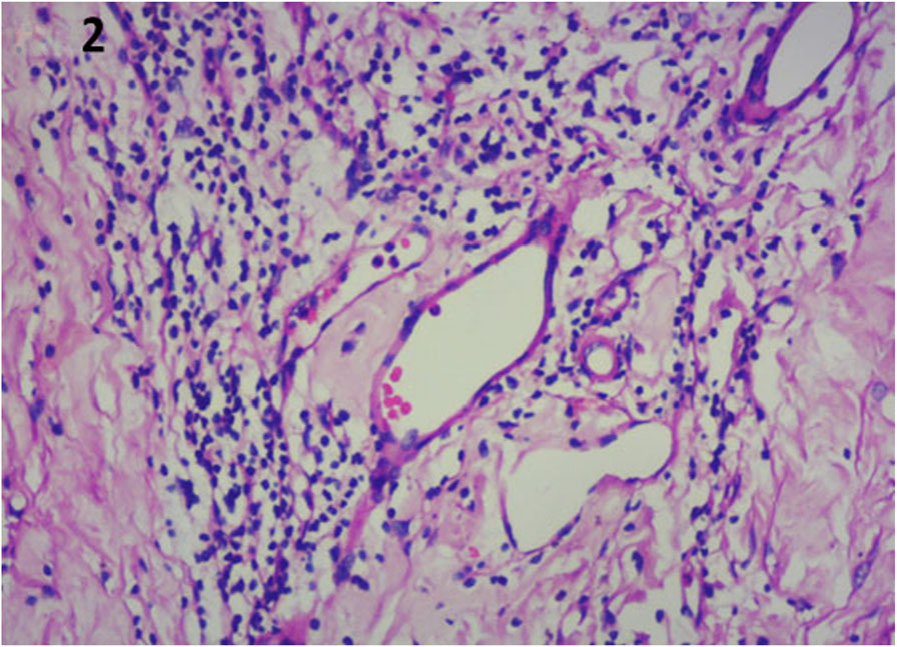
Histopathology section shows neovascularization into vitreous cavity with presence of lymphocytes in the inner vascular areas as well as perivascular spaces

**Fig. 3:**
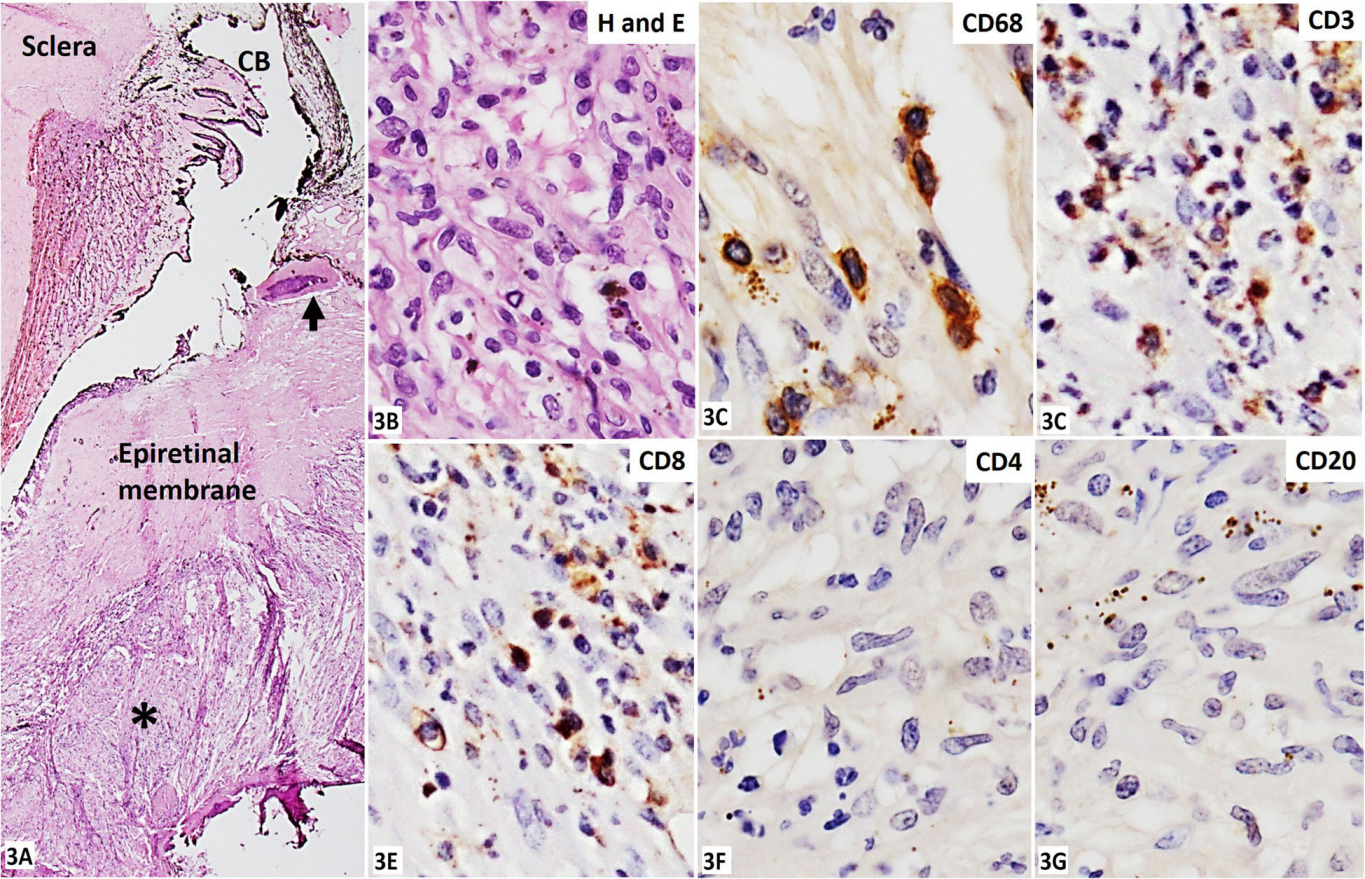
Scanner view of eyeball showing a thick epiretinal membrane which is densely fibrotic, scarred, relatively avascular and flanked above and below by metaplastic bone (arrow). Inflammation is seen below the membrane (asterix) **[A]**. Higher magnification of inflammatory focus shows collections of epithelioid cells forming an ill formed granuloma **[B].** The epithelioid histiocytes are CD68+ **[C]** and lymphoid cells are predominantly CD3+ **[D]**, CD8+ cytotoxic in phenotype **[E]** without CD4+ T cells **[F]** and CD20+ B cells **[G]**. (Magnification **A**: H&E obj.× 4, **B**: H&E obj.× 20, **C**: CD68 obj.× 20, **D**: CD3 obj.× 20, **E**: CD8 obj.× 20, **F**: CD4 obj.× 20, **F**: CD20 obj.× 20)

**Fig. 4:**
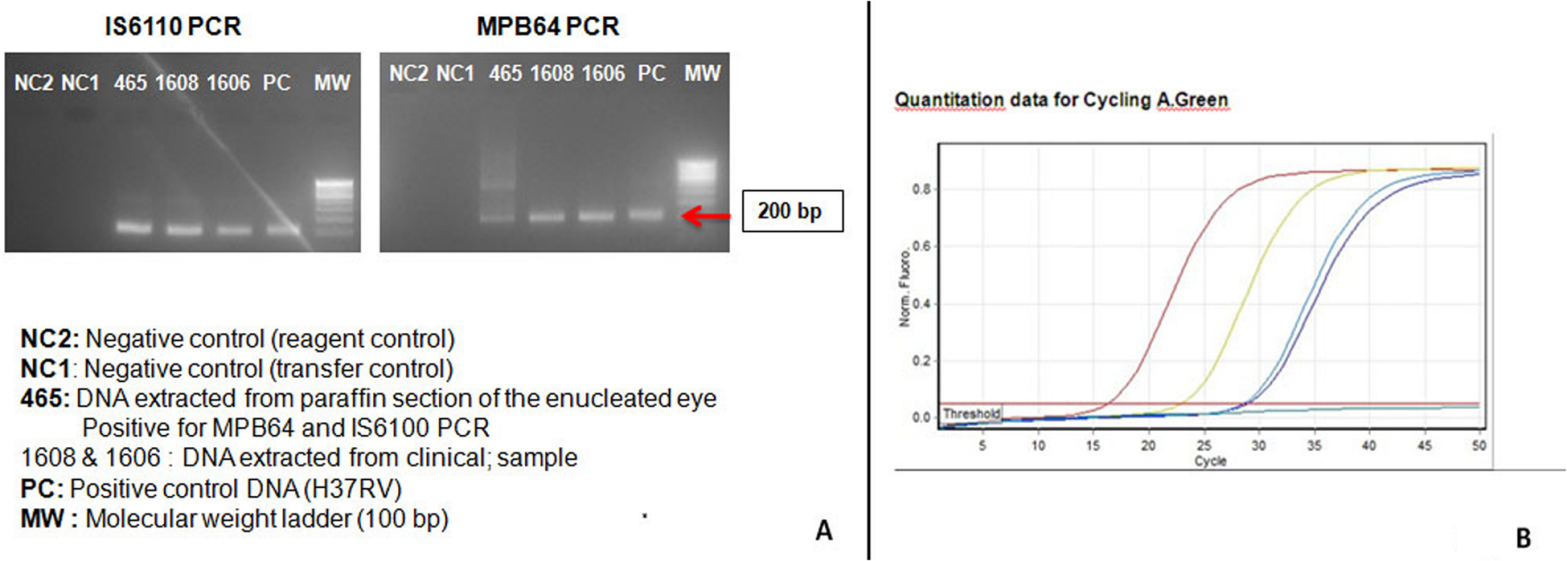
**A** Nested PCR for DNA extracted from paraffin section of the enucleated eye positive for MPB64 and IS6110. **B:** Real time PCR of DNA from paraffin section of enucleated globe Blue) showing 3460 copies

A 29-year-old Asian Indian male was diagnosed as Eales’ disease in the left eye and was referred to the uvea clinic of our tertiary eye care center in south India for persistent inflammation in the left eye. He was treated with an oral steroid and had pan-retinal laser photocoagulation of neovascularisation of retina. He had poor vision in the right eye since 10 years. On examination, his best corrected visual acuity was no perception light in the right eye and 20/200; N36 in the left eye. Right eye was phthisical with pupillary membrane, peripheral anterior and posterior synechiae, complicated cataract and low intraocular pressure. Anterior segment of left eye was normal. Indirect ophthlomoscopy of the fundus showed media haze at the posterior pole, a pale optic disc with attenuated and sclerosed vessels. There was few retinal hemorrhages along with collaterals temporal to the macula. Midperipheral retina showed laser photocoagulation scars (Fig. 1A). Fundus fluorescein angiography in the arterio-venous phase showed disc leakage with active vasculitis temporal to fovea (Fig. 1B). Ischemic areas were also noted temporal to macula. Fundus lesions were suggestive of active Eales’ disease in the left eye. The patient was investigated for causes of retinal vasculitis. Laboratory investigations for toxoplasma, syphilis, sarcoidosis, and collagen diseases were negative. The right eye was enucleated for cosmetic reasons. The eyeball was fixed in 10% neutral buffered formalin, sectioned axially and subjected to processing for paraffin embedding. Serial sections were stained with Hematoxylin and Eosin, Masson’s trichrome stain for collagen and Ziehl-Neelsen stain (ZN) for acid fast bacilli. IHCs was performed for multiple markers, which included glial fibrillary acidic protein (GFAP), CD45, CD68, CD3, CD4, CD8, CD20, CD138, MPO, IgG and IgG4. Histopathology revealed a phthisic eyeball with a thick epiretinal membrane and retinal detachment. The epiretinal membrane was densely collagenized and scarred, relatively avascular with sclerotic vessels reflecting chronicity and flanked above and below by metaplastic bone. A think band of dense fibrillary gliosis was seen in the adherent retina. Inflammation was seen forming a small aggregate beneath the ciliary body. It had an admixture of lymphocytes, histiocytes with elongated nuclei and epithelioid morphology forming loose clusters, admixed with lymphocytes and few polymorphs (Fig. 2). The lymphoid cells were CD3+ T cells with scant to absent CD20+ B cells. The T cells were CD8+ cytotoxic cells. No CD4 + T cells or plasma cells were seen. IgG4 was negative. (Fig. 3A-G). ZN stain for acid fast bacilli was negative. DNA was extracted from the paraffin section and nested PCR targeting MPB64 gene and IS6110 region of mycobacterium tuberculosis (MTB) genome were found to be positive (Fig. 4A). Real time PCR showed 3460 of copies of MTB DNA (Fig. 4B). Patient was put on four drug antitubercular treatment with oral Prednisolone 60 mg per day,which was gradually tapered over 6 weeks. On last follow up, there was complete resolution of vasculitis. However, there was no improvement of vision in OS due to macular ischaemia.

## Discussion

Eales disease is a retinal vasculitis characterized by retinal inflammation, ischaemia and neovascularization [1]. There were only few histopathologic studies on Eales’ disease eye performed [3]. PUBMED search did not show any IHCs studies of Eales’ diseases so far. Our study showed cytotoxic T-cells in the eye ball specimen of Eales disease. T cell mediated immunologic reaction has been found in Behcet’s disease [4], Vogt Koyanagi Harada disease [5] and sympathetic ophthalmia [6]. Our study indicates in active stage of Eales disease, immunomodulatory therapy with T cell inhibitors may be effective. Eales disease is thought to be an immunologic reaction that can be triggered by MTB DNA. We had also found TB DNA earlier in the paraffin retrieval sections of enucleated globes in Eales disease [1]. Molecular biologic study of the vitreous sample [7] as well as epiretinal membrane [8] of Eales’ disease by PCR showed MTB DNA. We had also found earlier MTB DNA in the paraffin retrieval sections of enucleated globes in Eales disease [3].

Our present study showed for the first time that Eales’ disease is associated with MTB by nested and real time PCR of the enucleated specimen correlated with histology and IHCs. IHC finding predominantly showed CD8 + T cells lymphocytic infiltration in the enucleated specimen. Granulomatous response associated with MTB is usually type IV hypersensitivity mediated by CD4 + T cells. In contrast in Eales disease, we observed a CD8 + T cell response which could be attributed to the ocular immune privilege. Targeted immunomodulation directed against CD8 + T cells could aid in specific therapy. We conclude that Eales’ disease is probably a cytotoxic T cell (CD8) mediated process which is triggered by mycobacterial MTB DNA. However, further studies are needed to confirm our findings.

## Data Availability

Yes, available.
